# Cervical spine involvement in rheumatoid arthritis over time: results from a meta-analysis

**DOI:** 10.1186/s13075-015-0643-0

**Published:** 2015-05-31

**Authors:** Tony Zhang, Janet Pope

**Affiliations:** Schulich School of Medicine & Dentistry, Western University of Canada (formerly University of Western Ontario), St. Joseph Health Care, 268 Grosvenor Street, London, ON N6A 4 V2 Canada

## Abstract

**Introduction:**

Complications in rheumatoid arthritis (RA) seem less common than they were years ago. The prevalence and progression of anterior atlantoaxial subluxations (aAASs), vertical subluxations (VSs), subaxial subluxations (SASs), and associated cervical myelopathy in RA over the past 50 years were determined.

**Methods:**

A literature search was performed by using Medline-OVID/EMBASE, PubMed, and Scopus (from 1960 to June 21, 2014). Prevalence studies were included if the sample size was at least 100 or the prevalence/progression of cervical subluxations was reported. Study quality was assessed by using the Strengthening the Reporting of Observational Studies in Epidemiology (STROBE) checklist. Prevalence of cervical subluxations was calculated for each study. Student’s *t* test and meta-regression were used to evaluate for significance.

**Results:**

In total, 12,249 citations were identified and 59 studies were included. The prevalence of aAAS decreased from 36% (95% confidence interval (CI) 30% to 42%) before the 1980s to 24% (95% CI 13% to 36%) in the 2000s (*P* = 0.04). The overall prevalence rates were 11% (95% CI 10% to 19%) for VS, 13% (95% CI 12% to 20%) for SAS, and 5% (95% CI 3% to 9%) for cervical myelopathy, and there were no significant temporal changes. Rates of progression of aAAS, VS, and SAS were 4, 6, and 3 lesions per 100 patients per year, respectively. The incidence of new or progressive cervical myelopathy was 2 cases per 100 patients with known cervical subluxations per year.

**Conclusions:**

Since the 1960s, only aAAS has decreased dramatically. It is still more than twice as common as VS or SAS. No temporal changes in the development of cervical myelopathy in affected patients with RA were noted. The progression rates of cervical subluxations and myelopathy were unchanged over time.

## Introduction

Rheumatoid arthritis (RA) is a chronic systemic inflammatory disease that primarily affects the joints. Although inflammatory arthritis of the small joints in the hands and feet is a common clinical manifestation, the cervical spine can also be affected. In fact, cases surfaced as early as 1890, when A. E. Garrod reported 178 patients with cervical spine involvement in a series of 500 patients with RA [1]. Cervical spine involvement can take many forms. Reported findings include vertebral endplate erosions, spinous process erosions, and apophyseal joint changes, such as osteoporosis, blurring, and fusion [2,3]. The most characteristic lesions are subluxations [4,5].

The atlas-axis—cervical vertebrae 1 and 2 (C1 and C2)—articulation is one of the prime disease targets. The erosive pannus formation at this joint often leads to bony destruction and laxity in the surrounding ligamentous complex, especially the transverse ligament that aligns that atlas and axis. The subsequent loss of anchoring structures results in atlantoaxial subluxation (AAS) [6]. The subluxation can be anterior, posterior, lateral, and rotatory. The anterior atlantoaxial subluxation (aAAS) is the most common subtype; the reported prevalence ranges from 10% to 55% [2,3,7-9]. This wide range may be related to the variation in the radiographic definition of aAAS. Separation of the anterior odontoid peg (dens) from the anterior ring of atlas (altanto-dental interval) of greater than 2.5 mm to 5 mm has been considered pathological [2,10-12]. Posterior AASs are less frequent and often due to fracture of the dens [13]. Lateral subluxations are considered when lateral masses of C1 are displaced laterally more than 2 mm in comparison with C2. They can lead to head tilt and rotational deformities [5].

Another form of subluxation is the vertical subluxation of the axis (VS), also known as altantoaxial impaction, cranial settling, superior migration of the odontoid, or psuedobasilar invagination. It is secondary to the destruction of occipitoatlantal and atlantoaxial joints and surrounding soft tissues [5]. Not surprisingly, many studies have shown that AAS and the destruction of C1-C2 articulation tend to predate the development of VS [14-16].

Lastly, subaxial subluxation (SAS) is associated with the damage to the facet joints, interspinous ligaments, and intervertebral discs. It can happen at one or multiple levels, leading to a step-ladder deformity and kyphosis [5]. SAS is defined by a misalignment of more than 3 mm between the anterior bodies of two consecutive vertebrae, although a threshold of 1 mm has also been found in studies [4,17]. Neck subluxations can develop over the course of the disease process, and there is an increased risk of developing neurological complications. Tinnitus, vertigo, and visual changes associated with vertebrobasilar insufficiency or brainstem compression can arise [6]. Patients may experience myelopathic symptoms. Once severe neurological complications develop, the prognosis can be grave; 1-year mortality can be as high as 50% if the condition is left untreated [18].

It appears that RA complications such as rheumatoid nodules, corneal melts, vasculitis, and C-spine subluxations are less frequent than they were years ago. Given the improved RA management over the past 50 years, our objectives were to quantify and evaluate temporal changes in the prevalence and progression of various RA-related cervical subluxations and cervical myelopathy.

## Methods

### Study selection

A comprehensive literature search was performed by using the Medline-OVID/EMBASE, PubMed, and Scopus databases from 1960 to June 21, 2014. We looked for studies of cervical subluxations in patients with RA by using the following search strategy:

1. rheumatoid arthritis
2. cervical vertebrae
3. atlanto-axial joint
4. spinal diseases
5. joint instability
6. AAS
7. atlantoaxial instability
8. atlantoaxial dislocation
9. VS
10. superior migration of the odontoid
11. cranial settling
12. pseudobasilar invagination
13. atlantoaxial impaction
14. SAS
15. or/2 to 14
16. 1 and 15.

Identified titles/abstracts were reviewed, and full reports were obtained if appropriate. Studies were considered if they provided data on the prevalence or progression of any cervical subluxations in patients with RA. Studies reporting prevalence required a sample size of at least 100 patients with RA. Another inclusion criterion was publication in English. Case reports, case series of fewer than 100 patients for the estimate of prevalence, and review articles were excluded. Cervical spine involvement of RA patients who were followed over time was included for the rate of progression analyses. Additional articles were retrieved by hand by searching relevant references.

### Data collection

Information from the studies was extracted by one reviewer (TZ) and ambiguities were resolved with the other reviewer (JP). A standard data extraction form was used to extract the following information:

Prevalence studies: year of publication, author, location of study, study design, patient population, sample size, number of females, mean age, mean disease duration, and proportion with any cervical changes, aAAS, posterior AAS, lateral AAS, rotatory AAS, VAS, SAS, cervical myelopathy, brainstem compression/vertebral artery involvement, and their respective definitions.

Progression studies: year of publication, author, location of study, study design, patient population, sample size, number of females, mean age, mean disease duration, years of follow-up, baseline, and rate of progression of aAAS, VS, SAS, cervical myelopathy and number of progressive/new cervical myelopathies. If studies included patients with RA and other rheumatic diseases, only data pertaining to the RA cohort were extracted.

### Quality assessment

Each study was assessed by using the Strengthening the Reporting of Observational Studies in Epidemiology (STROBE) checklist [19], which is a 22-item checklist relating to the title and abstract of the article (item 1), background and objectives (items 2 and 3), methods (items 4 to 12), results (items 13 to 17), discussion (items 18 to 21), and funding (item 22). The purpose of STROBE is not to give a quality score but to ensure clear presentation of reporting.

### Statistical analysis

Proportions were pooled with a random effects model [20]. Forest plots were created to estimate prevalence with a 95% confidence interval (CI). The I^2^ statistic was used to quantify the magnitude of heterogeneity (mild, 0% to 30%; moderate, 31% to 50%; high, more than 50%). Tau-squared was the square root of the between-study variance, and *P* value was for Cochrane’s Q, the classic measure of heterogeneity. Owing to significant heterogeneity, Forest plots and pooled estimates were not reported. Student’s *t* test was performed to evaluate regional differences in aAAS. Meta-regression through a random effects model was also used to explore the study-level associations between the prevalence of various C-spine complications and year of publication. A *P* value of less than 0.05 was considered statistically significant. Publication bias was determined by using funnel plots.

## Results

### Search results

**Figure 1:**
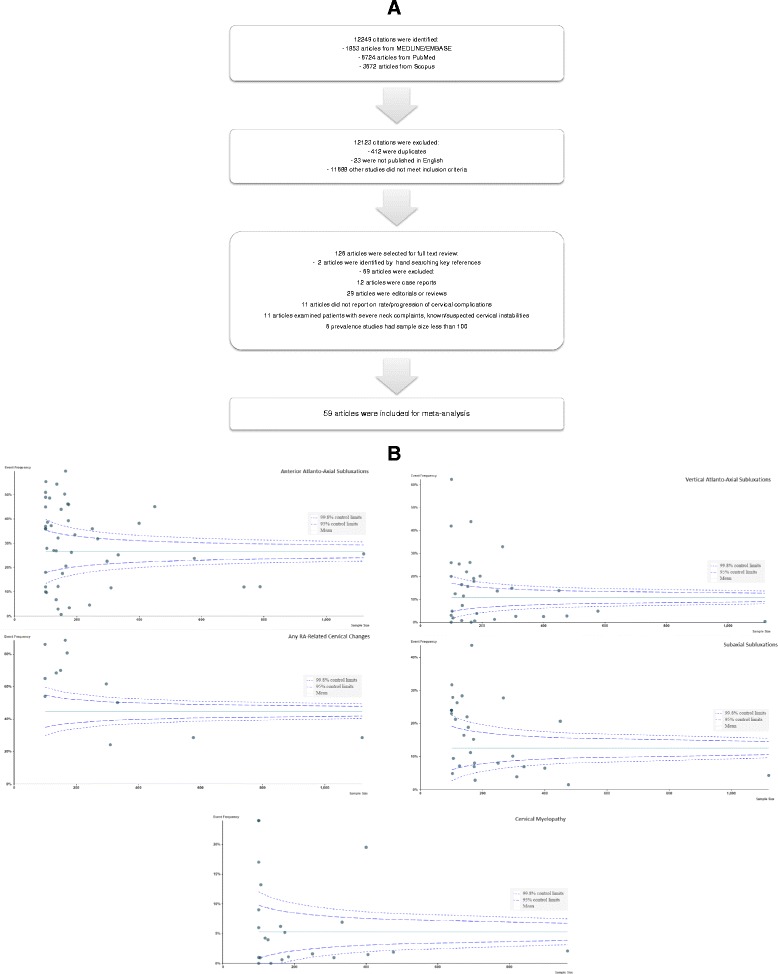
**(A)** Search strategy for articles and **(B)** Funnel plots to determine if there was publication bias. Funnel plot 1: Graph of sample size of individual study (x-axis) and its respective prevalence of anterior atlanto-axial subluxations (y-axis). Funnel plot 2: Graph of sample size of individual study (x-axis) and its respective prevalence of rheumatoid arthritis (RA)-related cervical changes (y-axis). Funnel plot 3: Graph of sample size of individual study (x-axis) and its respective prevalence of vertical atlanto-axial subluxations (y-axis). Funnel plot 4: Graph of sample size of individual study (x-axis) and its respective prevalence of subaxial subluxations (y-axis). Funnel plot 5: Graph of sample size of individual study (x-axis) and its respective prevalence of cervical myelopathy (y-axis).

The literature search identified 12,249 citations with 412 duplicates. Titles or abstracts (if available) were screened for eligibility, yielding 126 citations for full text review. Twelve studies were case reports. Twenty-nine studies were editorials or reviews. Eleven studies did not report on the rate or progression of cervical complications. Eleven studies examined patients with severe neck complaints or suspected/known cervical instabilities. Six excluded studies examined the prevalence of cervical instabilities in RA but had a sample size of less than 100. Two articles were identified by hand-searching key references. In total, 67 articles were excluded, leaving 59 studies to be included in our meta-analysis (Figure 1).

### Description of the included studies and participants

Fifty studies reported on the prevalence of cervical instabilities. Three studies were published in the 1960s with 533 participants (4% of pooled study population), nine studies in the 1970s with 2,798 participants (23%), nine studies in the 1980s with 1,449 participants (12%), nine studies in the 1990s with 1,653 participants (14%), 13 studies in the 2000s with 3,777 participants (31%), and seven studies in the 2010s with 2,012 participants (16%) [2-4,7-12,15,17,21-59]. The total number of enrolled patients was 12,222. Major world regions were represented. Six studies were from the United States, including 855 participants (7% of pooled study population) [3,15,17,21,33,35]. Twenty-six studies were from Europe with a total of 7,315 participants (60%) [2,4,7,10,22-25,27-31,34,37,38,40,43-46,49-52,59]. Sixteen studies were conducted in Asia, totaling 3,662 participants (30%) [8,9,12,26,32,39,41,42,47,48,53-58]. An additional study compared a Malaysian cohort with a British cohort with a total of 140 patients [36]. One study was from Africa and included 250 people (2%) [11]. The average female representation was 75%. The mean age at the time of outcome assessment was 58 years old (range of 33 to 69 years), and the mean disease duration was 12 years (range of 2 to 30 years).

Twenty studies were included for analyzing the progression of cervical instabilities. Data were obtained from 12 of the studies described above as well as nine additional studies [7,8,14-16,22,23,25,29,32,42,55,56,60-66]. The number of enrolled participants was 2,157, and the average follow-up was 6 years (range of 3 to 10 years). Eighty percent of the participants were female. The mean age and disease duration at the time of final assessment were 60 years (range of 50 to 67 years) and 15 years (range of 7 to 30 years), respectively.

Across all studies, the median number of items fulfilled on the STROBE checklist was 16 (range of 11 to 19) and reporting was improved in more recent studies. Characteristics of the included studies are shown in Tables 1 and 2.

**Table 1:** **Characteristics of 50 studies reporting prevalence of cervical subluxations**

**Year of publication**	**First author (reference)**	**Country**	**STROBE checklist (total = 22)**	**Sample size**	**Female, number (percentage)**	**Mean age, years**	**Disease duration, years**
1963	Bland [21]	USA	11	100	65 (65%)	56	14.4
1966	Conlon [2]	New Zealand	14	333	230 (69%)	52.7	N/A
1969	Park [3]	USA	12	100	N/A	N/A	10
1971	Meikle [4]	Scotland	15	118	83 (70%)	57.6	12.9
1971	Isdale [22]	New Zealand	13	171	N/A	N/A	12.1
1971	Stevens [10]	UK	16	100	73 (73%)	54.2	11.3
1972	Smith [23]	UK	13	962	588 (61%)	51.3	7.4
1975	Henderson [24]	UK	15	476	N/A	N/A	N/A
1976	Shaw [25]	UK	11	100	73 (73%)	54.2	N/A
1978	Higashiyama [26]	Japan	12	100	91 (91%)	N/A	N/A
1978	Chevrot [27]	France	14	577	N/A	N/A	N/A
1979	Jensen [28]	Denmark	14	194	N/A	N/A	N/A
1981	Winfield [29]	UK	14	100	56 (56%)	54.8	7.7
1981	Pellici [15]	USA	14	106	88 (83%)	62.6	30.1
1982	Halla [17]	USA	15	126	86 (68%)	57.6	11.1 s
1983	Winfield [7]	UK	17	100	57 (57%)	57.1	10.0
1984	Haaland [30]	Norway	16	104	77 (74%)	N/A	15.3
1985	Redlund-Johnell [31]	Sweden	16	450	351 (78%)	63.5	N/A
1987	Morizono [32]	Japan	14	100	92 (92%)	57.1	12.8
1989	Floyd [11]	South Africa	15	250	187 (75%)	52.8	N/A
1989	Collins [33]	USA	14	113	59 (52%)	55	15
1990	Kauppi [34]	Finland	17	164	136 (83%)	57.3	19.3
1990	Halla [35]	USA	16	310	198 (64%)	68.8	10.2
1993	Veerapen [36]	Malaysia Britain	18	140	140 (100%)	46.5	10.1
1994	Stiskal [37]	Austria	18	136	110 (81%)	57	13
1994	Montemerani [38]	Italy	17	183	147 (80%)	33.4	12.3
1996	Aggarwal [39]	India	16	100	73 (73%)	N/A	5
1998	Fujiwara [8]	Japan	17	173	146 (84%)	55.6	12.5
1998	Schramm [40]	Germany	14	150	134 (89%)	56.5	12.3
1999	Yoshida [41]	Japan	16	297	251 (85%)	57.0	13.0
2000	Fujiwara [42]	Japan	15	161	134 (83%)	59.5	16.5
2000	Neva [43]	Finland	16	176	111 (63%)	48.5	2.5
2001	Riise [44]	Norway	16	241	156 (65%)	61.9	5.4
2003	Carmona [45]	Spain	18	788	562 (71%)	61.0	10.0
2003	Neva [46]	Finland	17	103	70 (68%)	56.0	9.8
2004	Mitsuka [47]	Japan	15	174	156 (90%)	60.9	19.1
2004	Pisitkun [48]	Thailand	17	134	124 (93%)	48.9	5.0
2004	Naranjo [49]	Spain	16	736	530 (72%)	61.4	9.0
2005	Zikou [50]	Greece	17	165	143 (87%)	59.6	12.3
2005	Vesela [51]	Czech Republic	15	400	N/A	N/A	N/A
2006	Neva [52]	Finland	18	154	117 (76%)	62.0	16.0
2008	Kim [53]	Korea	18	405	365 (90%)	56.0	10.8
2009	Al-Ghamdi [54]	Saudi Arabia	18	140	105 (75%)	47.2	6.9
2010	Imagama [12]	Japan	17	100	84 (84%)	61.0	13.0
2010	Ahn [55]	Korea	16	1,120	N/A	N/A	N/A
2011	Yurube [56]	Japan	18	267	220 (82%)	66.9	N/A
2012	Hirano [9]	Japan	18	101	N/A	65.6	19.2
2012	Yurube [57]	Japan	19	140	107 (76%)	68.3	18.5
2012	Eser [58]	Japan	17	150	126 (84%)	53.2	12.3
2013	Blom [59]	The Netherlands	16	134	90 (67%)	60.6	9.5

N/A, not applicable; STROBE, Strengthening the Reporting of Observational Studies in Epidemiology.

**Table 2:** **Characteristics of 20 studies reporting progression of cervical subluxations**

**Year of publication**	**First author (reference)**	**Country**	**STROBE checklist (total = 22)**	**Sample size**	**Female, number (percentage)**	**Mean age, years**	**Disease duration, years**	**Years of follow-up**
1971	Isdale [22]	New Zealand	13	171	N/A	N/A	12.1	6.0
1972	Smith [23]	UK	13	84	64 (76%)	N/A	N/A	7.8
1974	Matthews [14]	UK	11	54	N/A	N/A	N/A	5.0
1976	Shaw [25]	UK	11	75	N/A	N/A	N/A	6.0
1981	Winfield [29]	UK	14	100	56 (56%)	54.8	7.7	7.2
1981	Pellici [15]	USA	14	106	88 (83%)	62.6	30.1	6.1
1982	Weissman [60]	USA	16	109	90 (83%)	63.1	20.9	4.9
1983	Winfield [7]	UK	17	100	57 (57%)	57.1	10.0	9.5
1987	Morizono [32]	Japan	14	27	27 (100%)	56.3	22.4	5.3
1989	Rana [16]	USA	15	41	N/A	N/A	N/A	10
1995	Oda [61]	Japan	14	49	43 (88%)	53.8	10.0	7.8
1997	Paimela [62]	Finland	16	67	56 (84%)	49.7	7.0	6.5
1997	Fujiwara [63]	Japan	13	79	66 (84%)	N/A	N/A	6.4
1998	Fujiwara [8]	Japan	17	173	146 (84%)	55.6	12.5	5.9
2000	Fujiwara [42]	Japan	15	161	134 (83%)	59.5	16.5	10.2
2010	Ahn [55]	Korea	16	137	123 (90%)	55.1	7.4	3.0
2011	Yurube [56]	Japan	18	267	220 (82%)	66.9	N/A	6.0
2012	Kaito [64]	Japan	18	38	33 (87%)	61.0	14.2	4.4
2013	Kaito [65]	Japan	18	91	74 (81%)	60.7	14.9	3.9
2014	Yurube [66]	Japan	19	228	183 (80%)	67.0	19.2	6.0

N/A, not applicable; STROBE, Strengthening the Reporting of Observational Studies in Epidemiology.

### Prevalence of any rheumatoid arthritis-related changes, cervical subluxations, and cervical myelopathy

Twelve studies reported the prevalence of any RA-related cervical changes, not limited to subluxations. Out of 3,559 patients, 1,597 (45%) were found to have one or more of those radiographic changes. There were no significant changes over time of publication (*P* = 0.71).

AAS was the most common subluxation found in our study. Twenty-seven percent (2,737 patients) had aAAS. Lateral AAS was reported in 68 patients (0.7%). Posterior and rotatory AASs were found in 21 (0.2%) and 10 (0.1%), respectively. In comparisons among different world regions, the prevalences of aAAS were 25%, 27%, and 31% for Europe, North America, and Asia, respectively (*P* = 0.13). The prevalences of aAAS were 36% in the 1970s and earlier (95% CI 30% to 42%), 36% in the 1980s (95% CI 23% to 42%), 32% in the 1990s (95% CI 20% to 44%), and 24% in the 2000s (95% CI 13% to 36%) (*P* = 0.04). VS and SAS had comparable prevalence rates. VSs were reported in 831 of 7,675 patients with RA with a prevalence of 11% (95% CI 10% to 19%); SAS occurred in 838 of 6,672 patients with RA (13%; 95% CI 12% to 20%). No significant temporal changes were noted for either VS or SAS (*P* = 0.17 and *P* = 0.49, respectively).

Twenty-three studies with 5,106 patients with RA reported on cervical myelopathy. Two hundred seventy-one patients (prevalence of 5%) had observed neurological deficits corresponding to Ranawat Cervical Myelopathy Classification II and above (95% CI 3% to 9%) [67]. We did not observe a significant change over time (*P* = 0.22). In terms of serious neurological complications, nine patients (0.2%) were observed to develop paraplegia. Ten additional patients (0.2%) had symptoms of possible brainstem involvement, including nausea, vertigo, or drop attacks.

### Progression of cervical subluxations and cervical myelopathy

Sixteen studies reported on the progression of 945 aAAS lesions. After 10,046 person-years, 219 of the existing subluxations had an increase in altanto-dental interval on radiography. Therefore, on average, 4 out of 100 existing aAAS lesions progressed per year. As for VS, 148 of 407 known lesions had radiographic progression, following 8,468 person-years. This corresponded to a progression rate of 6 per 100 lesions per year. Lastly, only 5 studies reported on SAS progression. A total of 46 out of 208 existing lesions progressed during the 4,781 person-years of follow-up. Therefore, the rate of SAS progression was 3 lesions per 100 existing lesions per year. The rate of progression for these subluxations was not found to be significantly different over time (aAAS, *P* = 0.99; VS, *P* = 0.49). Owing to the small number of studies, the rate of SAS progression over time was not analyzed.

Cervical myelopathy was assessed in 1,310 patients with RA with 9,131 person-years of follow-up. A total of 772 people had AAS, VS, SAS, or a combination of subluxations. Seventy-nine had new or progressive cervical myelopathy at the end of follow-up. We thus calculated a rate of 1.5 new/progressive cervical myelopathies per 100 patients with known cervical subluxations per year. Over time, the rates were 0.8, 2.1, 1.3, and 2.4 for the 1970s, 1980s, 1990s, and 2000s, respectively (*P* = 0.05).

## Discussion

Cervical spine involvement in RA has long been recognized. Varying degrees of prevalence have been reported as reflected in our literature search. This can be explained by numerous factors, such as differences in sample size, disease duration, follow-up period, medications used, and radiological criteria. There may also be reporting bias in which once something is well described, fewer publications follow over time. To the best of our knowledge, this is the first study to systematically evaluate the prevalence and progression of cervical subluxations and cervical myelopathy in patients with RA.

The earliest form of cervical instability in patients with RA has been shown to be a reducible AAS [62]. AAS is the most common cervical subluxation in RA. (AAS is twice as common as VS or SAS, and other types are very uncommon.) No regional differences in aAAS were found between Asia, Europe, and North America. However, a significant temporal reduction in the prevalence of aAAS exists, decreasing by one third from the 1960s and 1970s to 2000. The could reflect the changes in RA management (earlier introduction of DMARDs with higher doses and more combinations), or changing epidemiology of RA with fewer complications compared to years ago, and or reporting bias where the complications that are well described are less likely to be published over time so the contemporary rates may not be fully reported (publication bias).

The effects of DMARDs were addressed specifically in a number of studies. For example, Neva and colleagues recruited 198 early RA patients who were randomly assigned to either combination DMARDs (sulfasalazine, methotrexate, and hydroxychloroquine) or a single DMARD with or without prednisone [43]. After 2 years of follow-up, aAAS was found in 3% of the patients. None of the patients who were receiving combination therapies had aAAS. Lending further support to the role of DMARDs in preventing cervical involvement, biological agents were examined in two studies. A cohort of 38 early RA patients who received more than 2 years of continuous infliximab treatment in addition to methotrexate and prednisone was followed for 4 years [64]. Only one of the patients developed *de novo* AAS. In another study, a group of 91 patients who were on infliximab, etanercept, or tocilizumab was followed for 4 years [65] and three developed AAS.

Whereas the prevalence of aAAS seemed to be on the decline with the introduction of DMARDs, that of SAS and VS did not. This could be related to the natural progression of cervical subluxations. Our results demonstrated that, once formed, 4 out of 100 aAAS lesions would have an increase in atlanto-dental interval per year. After a certain point, AAS can have the appearance of stabilization or even improvement in the atlanto-dental interval on x-ray, signaling the beginning of VS. For example, Rana followed 41 AAS patients over a period of 10 years. Ten percent of the patients had less AAS secondary to new development of VS and upward translocation of odontoid process [16]. Smith and colleagues reported a similar phenomenon in 13% of their cases [23]. SAS generally develops at a later stage and is often seen after development of VS [68]. The lack of temporal reduction in the prevalence of VS and SAS may therefore be because DMARDs are less effective in preventing progression of existing lesions or due to a very low prevalence resulting in insufficient power to detect a change. When biologics were used, if there was C-spine subluxation, 79% to 80% progressed further [64,65]. We suspect that, eventually, the prevalence of VS and SAS will decrease as fewer *de novo* AASs are being formed.

Although cervical subluxations are common as illustrated above, their clinical presentation is often asymptomatic or associated with neck pain. Neurological complications are less frequent and tend to occur later in the disease course [69]. In our study, we reported a 5% prevalence rate for observed neurological deficits in patients with RA on the basis of the Ranawat grading system. Class I patients have pain but no neural deficits and therefore were not counted for our study. Class II patients have subjective weakness with hyperreflexia and dyssthesias. Class III patients have objective weakness and long tract signs. Class IIIA patients are ambulatory whereas IIIB patients are non-ambulatory [67]. Serious complications were rare, however. Paraplegia was reported in 0.2% of patients with RA, and symptoms of possible brainstem involvement, such as vertigo and drop attacks, were found in another 0.2%. We did not observe a decrease in the already-rare prevalence or progression of cervical myelopathy over time.

This study has limitations. Non-English studies were excluded. Although some asymmetries existed in our funnel plots, we do not believe that this represented major publication bias. Our data had significant heterogeneity. Age, disease duration, RA activity and severity, and treatments such as DMARDs and corticosteroids were not assessed. Estimates were generated at a population level. Inclusion criteria for some case series were not described in detail, and so the generalizability of the findings is unknown. We did not present the overall meta-analysis results, because heterogeneity (as measured by the I^2^ with respect to between-study variation) was high, but we could explore trial-level differences contributing to the heterogeneity (such as year of publication) by using meta-regression, which quantifies the effect of a study characteristic (publication year) on the overall effect of heterogeneity in the rate of C-spine subluxations. The review may be limited by variation in study quality, especially with the earlier publications having lower STROBE scores (as is often seen when comparing older with more recent studies due to different standards). Lastly, another factor to consider when interpreting the study is that cervical complications tend to develop over time. Even though our review included data from recent studies, the long-term effects of modern DMARDs (type and how we use them) on development and progression of cervical instabilities have yet to be adequately studied, and it could take years before differences in complications can be identified as C-spine complications could take years to develop or be prevented.

## Conclusions

Patients with RA are still at risk for cervical spine involvement. Over the past 50 years, we have found a significant decrease in the prevalence of aAAS, possibly due to improved disease control with modern DMARDs or changing epidemiology of RA. However, aAAS remains the most common lesion with a prevalence of 27%, more than twice as common as SAS or VS. The prevalence of cervical myelopathy did not change dramatically and remains at 5% among patients with RA. The rate of progression of cervical subluxations and myelopathy did not seem to be affected significantly by DMARDs.

## Abbreviations

aAAS: anterior atlantoaxial subluxation
AAS: atlantoaxial subluxation
CI: confidence interval
DMARD: disease-modifying anti-rheumatic drug
RA: rheumatoid arthritis
SAS: subaxial subluxation
STROBE: Strengthening the Reporting of Observational Studies in Epidemiology
VS: vertical subluxation
